# Spatio-temporal modeling of tuberculosis among Brazil’s elderly: a 20-year ecological and population-based study

**DOI:** 10.1186/s12879-026-13044-9

**Published:** 2026-03-28

**Authors:** Davi Rios do Nascimento, Sarah Fonseca Serpa, Adeilton Gonçalves da Silva Júnior, Sávio Luiz Pereira Nunes, Carlos Eduardo Lopes Pires, Gabriel de Jesus Gama dos Santos, Phoebe Silva van Emmerik, Hugo Gabriel Ferreira dos Santos, Rodrigo Feliciano do Carmo, Carlos Dornels Freire de Souza

**Affiliations:** 1 Health Situational Analysis Observatory, Federal University of the São Francisco Valley (UNIVASF), Petrolina City, Pernambuco Brazil; 2 Federal University of the São Francisco Valley, Petrolina City, Pernambuco Brazil

**Keywords:** Tuberculosis, Elderly, Epidemiology, Spatial analysis, Public health, Brazil

## Abstract

**Background:**

Tuberculosis (TB) remains a significant public health issue, with an increasing impact among the elderly population. In Brazil, population aging poses additional challenges to disease control.

**Objective:**

To analyze the socioepidemiological and clinical profile, as well as the temporal and spatial dynamics of tuberculosis among individuals aged 60 years or older in Brazil between 2001 and 2022.

**Methods:**

This ecological, population-based study used secondary data on TB cases among older adults reported to the Notifiable Diseases Information System (SINAN). Sociodemographic and clinical characteristics were described, and temporal trends were assessed using Joinpoint regression. Spatial dependence and clusters were analyzed using Global and Local Moran’s I statistics.

**Results:**

A total of 228,912 TB cases were reported among older adults during the study period. Most cases occurred in males (65.8%), individuals aged 60–69 years (57.9%), Black and Brown individuals (46.3%), and those with low or no formal education (53.6%). There was an average annual reduction of 2.37% in TB incidence among the elderly in the country. Of the 27 federative units, 21 showed a decreasing trend, particularly the Federal District (− 4.97%) and Goiás (− 4.03%). Six states showed a stationary trend (Acre, Roraima, Paraíba, Sergipe, Minas Gerais, and Espírito Santo), and none showed a significant increasing trend. Spatial analysis revealed a persistent concentration of high-priority municipalities, especially in the North and Northeast regions, which accounted for 25.4% of the cases.

**Conclusions:**

Tuberculosis among older adults in Brazil demonstrated an overall declining trend over the past two decades, although with marked regional inequalities and persistent spatial concentration in historically vulnerable territories. These findings underscore the need for targeted strategies for surveillance, diagnosis, and care in priority territories, considering the specificities of population aging.

**Clinical trial number:**

Not applicable.

## Introduction

Tuberculosis (TB) is an infectious disease transmitted through direct contact with individuals infected with *Mycobacterium tuberculosis* [1]. Even though it is a long-known and treatable disease, tuberculosis is still considered a public health problem. It is estimated that approximately one quarter of the global population is infected with the bacillus, a condition defined as Latent Tuberculosis Infection (LTBI), and that around 10 million people develop the disease each year, with an estimated 1 million deaths [2].

In response to the public health threat posed by the disease, the WHO launched the ‘End TB’ strategy, aiming to reduce the number of new TB cases by 90% and TB-related deaths by 95% between 2015 and 2035, paving the way for its elimination (defined as an incidence of less than one case per million population) [3]. By 2023, the estimated global decline in TB incidence was less than − 2% per year, highlighting the need to accelerate this decline to up to − 17% per year after 2025 in order to meet the 2035 targets [4].

Although tuberculosis affects all age groups, with higher prevalence still observed among the younger population, a shift in the TB case profile among older adults has become evident, with a progressive increase in various regions of the world [5]. This change in the pattern of illness may reflect the global population aging process. It is estimated that by 2030, the number of people aged 80 years or older will surpass the number of infants under one year of age [6].

In Brazil, the Elderly Statute defines older adults as individuals aged 60 years or older [7]. This age group accounted for 10.78% of the population in 2010 [8], and increased to 14.7% in 2022 [9], highlighting the rapid population aging process in the country. Older adults represent approximately 14% to 16% of reported tuberculosis cases and account for nearly 40% of tuberculosis-related deaths in Brazil, exhibiting high incidence rates that increase progressively with advancing age [10].

The course of TB illness in older adults also poses a challenge. This population presents a higher frequency of atypical manifestations of the disease, a greater number of adverse drug reactions, and a higher TB-related mortality rate compared to younger individuals [11]. It is estimated that, globally, around 30% of tuberculosis deaths occur in people aged 65 years or older [12].

In this context, the aim of this study was to analyze the sociodemographic and clinical profile, as well as the temporal and spatial dynamics of tuberculosis among the elderly population in Brazil between 2001 and 2022.

## Methods

### Study design, population and period

This is an ecological study using data on tuberculosis cases reported among individuals aged 60 years or older in Brazil from 2001 to 2022. An ecological study is an observational epidemiological study that analyzes aggregated population data [13]. All cases recorded in the Notifiable Diseases Information System (SINAN) for this age group during the study period were included, without additional exclusion criteria, in order to preserve the population-based nature of the analysis.

### Location

The study covered Brazil, its five macro-regions (North, Northeast, Central-West, Southeast, and South), and the 27 federative units, with analyses conducted at national, regional, and state levels. Brazil is the fifth largest country in the world by area, with a territorial extension of approximately 8.5 million km², a resident population of about 203 million people, and a population density of 23.86 inhabitants per km² [9].

**Fig. 1:**
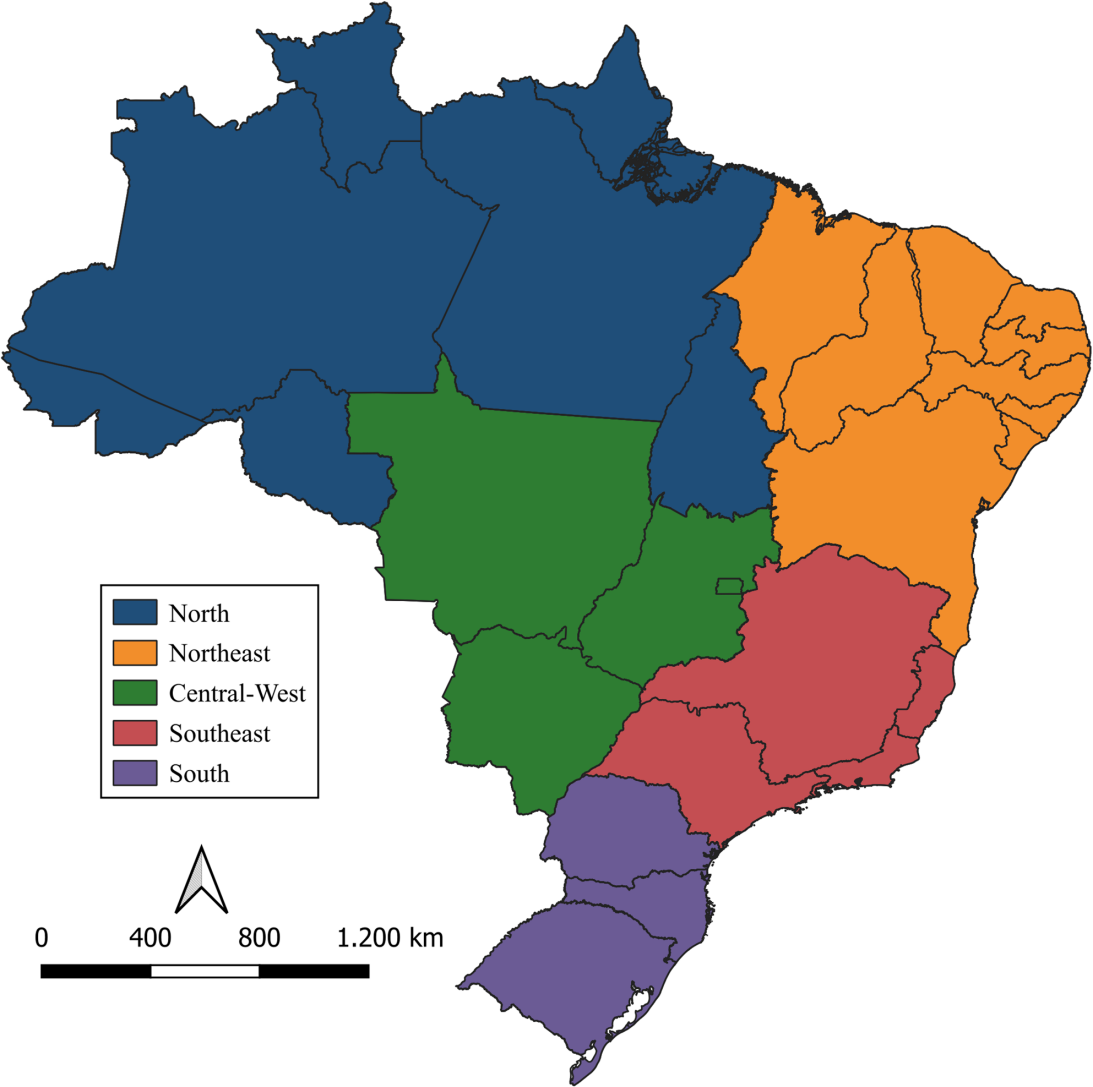
Geographic location of Brazil by macro-regions. The map highlights the five Brazilian macro-regions and federative units, providing spatial context for the analyses. Source: IBGE. Map prepared by the authors using QGIS

Figure 1 presents the geographic location of Brazil and its macro-regions and federative units, providing spatial context for the analyses and facilitating interpretation for international readers.

### Data source and variables

Data were obtained from the SINAN, the Brazilian national compulsory disease surveillance system. All variables analyzed in this study were extracted directly from the official tuberculosis notification forms used by SINAN, which standardize the collection of sociodemographic and clinical information nationwide. These forms, including variable definitions and coding schemes, are publicly available through the SINAN portal (https://portalsinan.saude.gov.br/tuberculose). The data were accessed in aggregated and anonymized form via the TabNet platform, maintained by the Department of Informatics of the Brazilian Unified Health System (DATASUS) (https://datasus.saude.gov.br/informacoes-de-saude-tabnet/).

The municipality of residence at the time of notification was used as the territorial unit of analysis. Territorial boundaries of Brazil, its macro-regions, federative units, and municipalities, as well as population estimates used to calculate tuberculosis incidence rates, were obtained from official Brazilian Institute of Geography and Statistics (IBGE) population censuses and intercensal projections, available at https://www.ibge.gov.br/.

Sociodemographic variables included age group (60–69, 70–79, and ≥ 80 years), sex, race/skin color (White, Black, Brown, Yellow, Indigenous, and Unknown), and education level. Education was categorized according to standard SINAN classifications and, for analytical purposes, grouped as < 8 years of schooling, ≥ 8 years of schooling, and unknown/blank. Clinical variables comprised type of entry, clinical form of tuberculosis, and site of extrapulmonary involvement, as recorded in the SINAN notification forms.

Treatment outcome was not included due to the study’s focus on incidence and spatiotemporal patterns and the high degree of incompleteness and temporal heterogeneity of this variable in SINAN. Records classified as “ignored,” “unknown,” or “post-mortem” were retained in the descriptive analyses as specific categories, in accordance with recommendations for the use of secondary data from epidemiological surveillance systems, and accounted for a small proportion of cases without substantive impact on temporal or spatial trends.

#### Stage 1 - Descriptive analysis of the socioepidemiological profile

In the first stage, a descriptive analysis of tuberculosis cases among older adults was conducted. Sociodemographic variables, as defined in the preceding section, included age group, sex, race/skin color, and education level. Clinical variables were analyzed separately and comprised type of entry, clinical form of tuberculosis, and site of extrapulmonary involvement, when applicable. Variables were summarized using absolute frequencies and relative proportions (%).

#### Stage 2 - Time series analysis

The incidence rate was calculated using the following formula.

$$\begin{aligned}&Incidence\:rate\cr&\quad=\frac{\begin{aligned}&(number\:of\:reported\:cases\:classified\cr&\:as\:"new\:cases,\:"\:"unknown,\:"or\:\cr&"post-mortem\:"inindividuals\:aged\cr&\:60\:years\:or\:older\:in\:the\:location)\end{aligned}}{\begin{aligned}&(population\:aged\:60\:years\:or\:older\cr&\:residing\:in\:the\:same\:period)\end{aligned}}\cr&\quad\times100,000\end{aligned}$$

To identify the temporal trend of TB incidence in older adults, the Joinpoint Regression model was used. This method evaluates whether a segmented line with multiple joinpoints better describes the data evolution over time than a continuous line or one with fewer segments. In addition to assessing the trend direction (stationary, increasing, or decreasing), the model identifies the joinpoints and estimates the Annual Percent Change (APC) and the Average Annual Percent Change (AAPC) for the entire period.

The following parameters were used: minimum of 0 and maximum of 2 joinpoints; model selection based on 4,499 permutations; 5% significance level; 95% confidence interval; and correction for residual autocorrelation. Analyses were performed using the Joinpoint Regression Program version 5.2.0 (National Cancer Institute, Bethesda, MD, USA). Modeling was applied to three spatial units: Brazil, macro-regions, and federative units.

#### Stage 3 - Spatial analysis

Spatial autocorrelation was assessed using the Global Moran’s I, aimed at identifying the presence of spatial dependence among municipalities. When such dependence was confirmed, the Local Moran’s I (Local Indicators of Spatial Association - LISA) was applied [14]. In this analysis, adjacent municipalities were considered first-order neighbors, with equal weights assigned to all adjacent units.

Based on the LISA results, municipalities were classified according to the quadrants of the Moran scatterplot:

- Q1 (high-high): municipalities with high indicator values surrounded by neighbors also with high values;
- Q2 (low-low): municipalities and neighbors with values below the overall mean;
- Q3 (high-low): municipalities with high values surrounded by neighbors with low values;
- Q4 (low-high): municipalities with low values surrounded by neighbors with high values.

For prioritization purposes, Q1 clusters were considered high priority, while Q2 and Q3 were classified as intermediate priority. The classification of incidence rate intervals displayed in the thematic maps was defined for descriptive and visualization purposes, using fixed and interpretable categories to facilitate comparison across regions and over time. The spatial analyses were conducted using QGIS (version 3.40.0; Open Source Geospatial Foundation, Beaverton, OR, USA) and GeoDa (version 1.22.0.10; Spatial Analysis Laboratory, Anselin, USA).

The spatial unit of analysis comprised all Brazilian municipalities officially recognized during the study period, totaling 5,570 municipalities. Municipal boundary files were obtained from the official cartographic database of the IBGE. Changes in municipal boundaries over time, including the creation of new municipalities, territorial dismemberments, or name changes between 2001 and 2022, were handled by adopting the most recent standardized municipal grid provided by IBGE, ensuring spatial consistency across the entire time series. This approach minimizes bias related to administrative changes and is commonly used in long-term ecological and spatial analyses.

Although socioeconomic indicators such as the Human Development Index (HDI) and the Social Vulnerability Index (SVI) are recognized as important determinants of tuberculosis distribution, a bivariate Moran’s I analysis was not performed in this study. This decision was based on the study’s primary objective of describing the spatiotemporal dynamics of tuberculosis incidence and on the limited temporal availability and comparability of these indices over the full study period. Nonetheless, the relationship between tuberculosis incidence and social vulnerability was addressed in the discussion, supported by previous national and international evidence.

### Ethical aspects

This study used only secondary data from public domain information systems, in which individuals cannot be identified. Therefore, approval from a Research Ethics Committee was not required.

## Results

Between 2001 and 2022, 228,912 elderly individuals were diagnosed with tuberculosis in Brazil, with higher occurrence among elderly males (65.79%; *n* = 150,606), those aged between 60 and 69 years (57.87%; *n* = 132,480), Brown and Black individuals (46.27%; *n* = 105,913), and those with low or no education (53.55%; *n* = 122,575). Regarding the clinical profile of the cases included in the analysis, 97.70% (*n* = 223,639) were classified as new cases according to SINAN records, reflecting the composition of the study database and the notification process rather than the overall epidemiological distribution of tuberculosis among older adults in Brazil. Among these notifications, pulmonary tuberculosis accounted for 84.00% (*n* = 192,297) of cases (Table 1).

**Table 1:** Sociodemographic and clinical distribution of tuberculosis cases among older adults reported in Brazil (2001–2022)

Sociodemographic variables	Number	%
**Age group**		
60–69	132,480	57.87%
70–79	68,484	29.92%
≥ 80	27,948	12.21%
**Sex**		
Male	150,606	65.79%
Female	78,268	34.19%
**Race/Color**		
White	79,472	34.72%
Black	22,529	9.84%
Brown	83,384	36.43%
Yellow	2,359	1.03%
Indigenous	2,517	1.10%
Unknown	38,629	16.88%
**Education level**		
< 8 years of schooling	122,575	53.55%
≥ 8 years of schooling	36,749	16.05%
Unknown/Blank	69,588	30.40%
**Clinical variables**		
**Type of entry**		
New case	223,639	97.70%
Unknown	3,356	1.47%
Post-mortem	1,917	0.84%
**Clinical form**		
Pulmonary	192,297	84.00%
Extrapulmonary	30,446	13.30%
Pulmonary + Extrapulmonary	6,056	2.65%
Unknown/Blank	113	0.05%
If extrapulmonary TB*		
Pleural	14,663	40.17%
Peripheral lymph node	4,764	13.05%
Other	4,464	12.23%
Bone	3,192	8.74%
Miliary	3,118	8.54%
Laryngeal	1,853	5.08%
Genitourinary	1,590	4.36%
Ocular	1,543	4.23%
Meningoencephalic	1,422	3.90%
Cutaneous	751	2.06%
*Total*	*228.912*	*100.00%*

*The variable “if extrapulmonary TB” was analyzed exclusively among cases classified as extrapulmonary or pulmonary plus extrapulmonary tuberculosis (*n* = 36,502), as this field is completed only for these clinical forms in the SINAN notification system

Among cases with extrapulmonary or mixed tuberculosis, pleural involvement was the most frequent extrapulmonary site, accounting for 40.17% of notifications. This was followed by peripheral lymph node involvement (13.05%) and bone involvement (8.74%), highlighting the heterogeneous distribution of extrapulmonary manifestations among older adults. Other sites, including miliary, laryngeal, genitourinary, ocular, meningoencephalic, and cutaneous forms, were observed with lower frequencies. After restricting the analysis to cases for which this variable is applicable, the proportion of unknown or blank records was low, indicating adequate completeness and reliability of the information regarding extrapulmonary site classification in the SINAN database (Table 1).

**Fig. 2:**
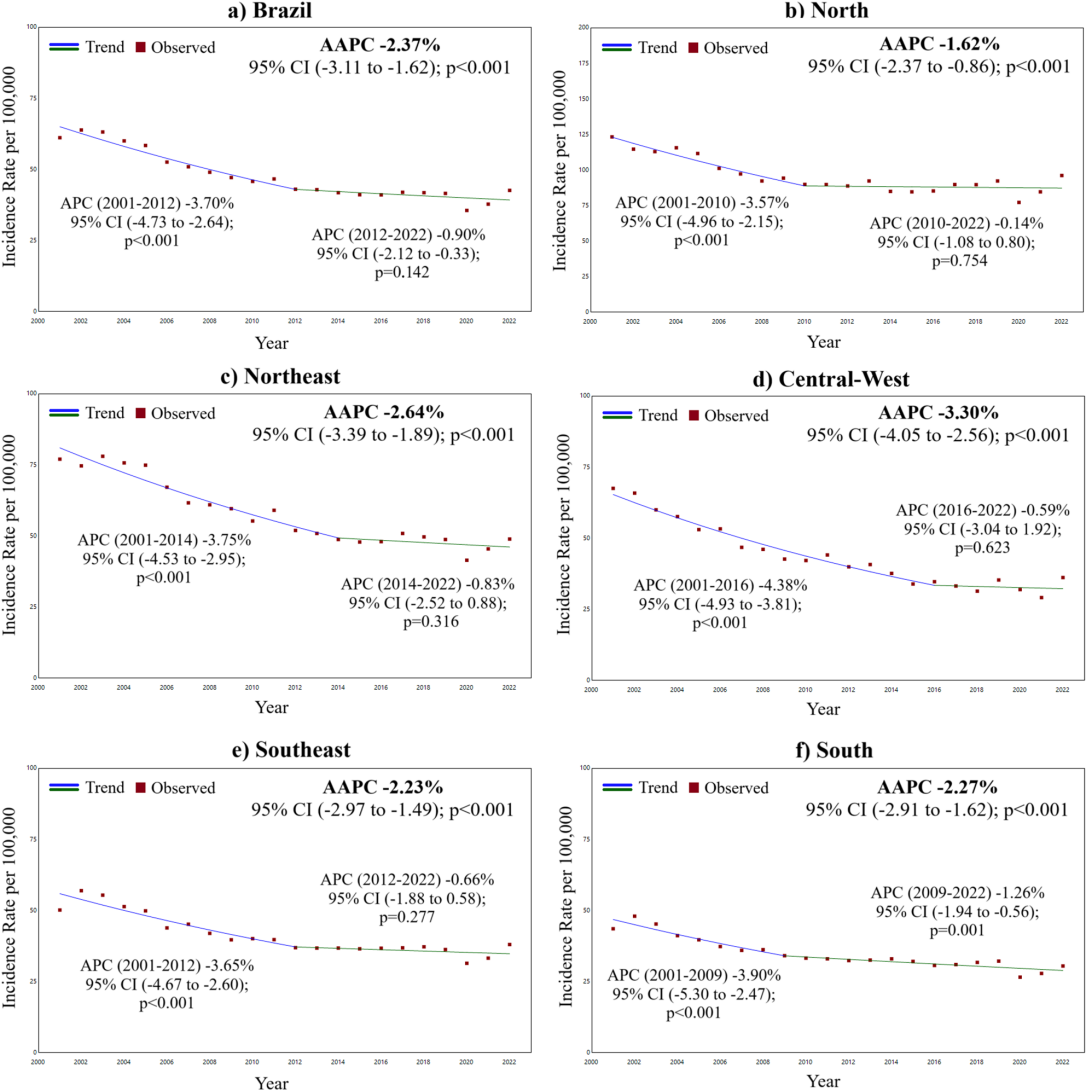
Joinpoint regression model for tuberculosis incidence rate among older adults in Brazil and regions, 2001–2022. AAPC: Average Annual Percent Change; APC: Annual Percent Change. The figure shows two joinpoints indicating a decreasing trend between 2001–2012 and a stationary trend from 2013–2022

An average annual decrease of 2.37% (95% CI: -3.11 to -1.62; *p* < 0.001) in the incidence rate of tuberculosis among the elderly in Brazil was observed between 2001 and 2022, declining from 61.08 cases per 100,000 inhabitants in 2001 to 42.56 cases per 100,000 inhabitants in 2022. All regions showed the same trend, with the Central-West region standing out with an average annual decrease of 3.30% (95% CI: -4.05 to -2.56; *p* < 0.001). This period presented two segments with one joinpoint: 2001 to 2012, with an annual decreasing trend of 3.70% (95% CI: -4.73 to -2.64; *p* < 0.001); and 2013 to 2022, with a stable incidence rate trend (APC − 0.90%; 95% CI: -2.12 to 0.33; *p* = 0.142) (Fig. 2).

The North and Northeast regions presented the highest average incidence rates (95.73 and 57.97 cases per 100,000 inhabitants, respectively) during the period, with Amazonas and Acre states standing out, with average incidence rates of 165.01 and 92.70 cases per 100,000 inhabitants. In addition to these, 15 other federative units presented average incidence rates above the national average: Rondônia; Roraima; Pará; Amapá; Maranhão; Piauí; Ceará; Rio Grande do Norte; Pernambuco; Alagoas; Bahia; Mato Grosso do Sul; Mato Grosso; Federal District; and Rio de Janeiro (Table 2).

**Table 2:** Average Annual Percent Change (AAPC) of the proportion of new tuberculosis cases in the elderly population evaluated by Incidence Rate, according to regions and federative units. 2001–2022

Spatial unit	Rate^1^					AAPC (CI 95%) *p* value	Trend
	2001		2022		Average Rate		
	Gross	Corrected	Gross	Corrected			
**North**	**123.21**	**122.99**	**96.02**	**87.19**	**95.73**	**-1.62% (-2.37 to -0.86;*** p* **< 0.001)**	↓
Rondônia	100.62	97.26	41.83	38.68	64.37	-4.29% (-5.03 to -3.54; *p* < 0.001)	↓
Acre	140.51	130.16	98.19	88.03	92.70	-1.84% (-4.24 to 0.61; *p* = 0.139)	↔
Amazonas	243.06	216.57	172.84	163.19	165.01	-1.33% (-2.82 to 0.16; *p* = 0.081)	↓
Roraima	127.55	95.03	95.12	78.64	89.69	-0.89% (-2.08 to 0.30; *p* = 0.133)	↔
Pará	92.80	100.79	92.43	78.50	89.52	-1.18% (-1.70 to -0.66; *p* < 0.001)	↓
Amapá	124.60	124.54	85.60	75.18	77.72	-2.37% (-4.58 to -0.11; *p* = 0.039)	↓
Tocantins	65.32	69.16	26.47	25.65	37.56	-4.61% (-6.76 to -2.41; *p* < 0.001)	↓
**Northeast**	**76.95**	**80.94**	**48.84**	**46.07**	**57.97**	**-2.64% (-3.39 to -1.89;*** p* **< 0.001)**	↓
Maranhão	110.18	110.33	63.99	59.56	76.29	-2.89% (-4.47 to -1.28; *p* < 0.001)	↓
Piauí	86.82	84.88	37.05	39.42	60.53	-3.58% (-5.93 to -1.18; *p* = 0.003)	↓
Ceará	72.96	76.96	56.10	46.82	60.90	-2.33% (-2.94 to -1.73; *p* < 0.001)	↓
Rio Grande do Norte	67.21	69.66	40.77	40.30	48.27	-2.57% (-3.77 to -1.35; *p* < 0.001)	↓
Paraíba	44.88	43.01	34.29	32.99	35.79	-1.25% (-4.67 to 2.29; *p* = 0.482)	↔
Pernambuco	70.97	76.45	58.78	53.82	62.07	-1.65% (-2.71 to -0.59; *p* = 0.002)	↓
Alagoas	53.30	58.71	42.52	39.01	48.39	-1.92% (-2.51 to -1.33; *p* < 0.001)	↓
Sergipe	34.26	32.94	38.70	35.60	40.68	0.37% (-4.00 to 4.94; *p* = 0.870)	↔
Bahia	90.80	85.42	43.90	38.16	59.03	-3.76% (-4.23 to -3.29; *p* < 0.001)	↓
**Central-West**	**67.48**	**65.33**	**36.12**	**32.22**	**43.74**	**-3.30% (-4.05 to -2.56;*** p* **< 0.001)**	↓
Mato Grosso do Sul	79.91	87.55	51.65	49.69	56.94	-2.66% (-4.05 to -1.24; *p* < 0.001)	↓
Mato Grosso	129.62	105.23	69.92	59.65	80.96	-2.66% (-3.52 to -1.79; *p* < 0.001)	↓
Goiás	49.73	49.83	22.19	20.99	27.89	-4.03% (-5.85 to -2.17; *p* < 0.001)	↓
Distrito Federal	26.24	44.22	16.43	15.14	27.63	-4.97% (-5.95 to -3.98; *p* < 0.001)	↓
**Southeast**	**50.13**	**55.87**	**37.98**	**34.75**	**41.37**	**-2.23% (-2.97 to -1.49;***p* **< 0.001)**	↓
Minas Gerais	8.49	14.55	22.81	18.55	28.76	1.16% (-2.05 to 4.48; *p* = 0.482)	↔
Espírito Santo	51.57	56.60	41.97	41.57	38.54	-1.45% (-4.05 to 1.21; *p* = 0.281)	↔
Rio de Janeiro	89.50	91.46	62.73	57.83	65.84	-2.15% (-2.79 to -1.51; *p* < 0.001)	↓
São Paulo	52.28	51.37	35.12	31.65	37.05	-2.27% (-3.09 to -1.45; *p* < 0.001)	↓
**South**	**43.52**	**46.79**	**30.41**	**28.87**	**34.86**	**-2.27% (-2.91 to -1.62;*** p* **< 0.001)**	↓
Paraná	43.22	44.90	20.55	20.29	28.31	-3.71% (-4.45 to -2.95; *p* < 0.001)	↓
Santa Catarina	27.74	29.13	25.08	23.58	26.37	-0.99% (-1.57 to -0.41; *p* = 0.001)	↓
Rio Grande do Sul	50.18	50.08	41.81	38.21	43.99	-1.27% (-1.74 to -0.81; *p* < 0.001)	↓
**Brazil**	**61.08**	**64.98**	**42.56**	**39.22**	**47.68**	**-2.37% (-3.11 to -1.62;***p* **< 0.001)**	↓

Statistical significance (*p* < 0.05); ^1−^ Rate/100,000 inhabitants; Gross rate: crude incidence rate per 100,000 inhabitants. Corrected rate: incidence rate adjusted for reporting inconsistencies over time

Average rate: mean annual incidence rate for the specified period; AAPC: Average Annual Percent Change; CI: confidence interval; ↓: decreasing; ↔: stationary; ↑ increasing

Between 2001 and 2022, 21 of the 27 federative units showed a decreasing trend in tuberculosis incidence rates among the elderly. In the Central-West and South regions, all states demonstrated this trend, with the Central-West region having the two units with the largest declines in the country: the Federal District and Goiás, with AAPCs of -4.97% (95% CI: -5.95 to -3.98; *p* < 0.001) and − 4.03% (95% CI: -5.85 to -2.17; *p* < 0.001), respectively. The remaining states showed stationary trends: Acre and Roraima in the North; Paraíba and Sergipe in the Northeast; and Minas Gerais and Espírito Santo in the Southeast (Table 2).

**Fig. 3:**
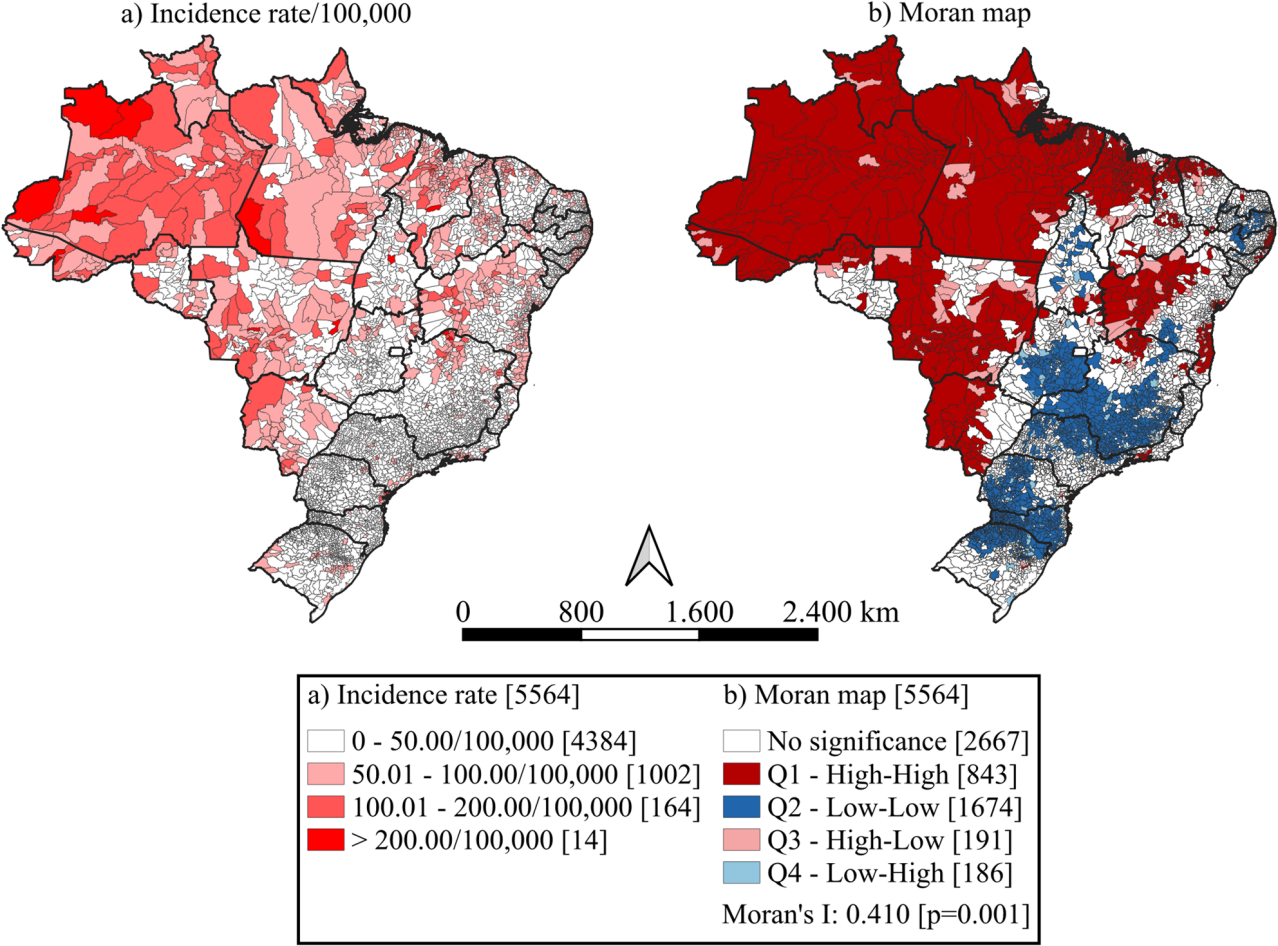
Spatial distribution of tuberculosis incidence rate and Moran’s I clusters among older adults, Brazil, 2001–2022. High-high clusters represent municipalities with high incidence rates surrounded by neighbors also with high rates. These priority areas were mainly concentrated in the North and Northeast regions

Regarding spatial analysis, a heterogeneous distribution of tuberculosis incidence rates among the elderly was observed during the study period, with spatial concentration predominantly in the North region, although there was expansion towards the Central-West and Northeast regions. A total of 78.8% (*n* = 4,384) of municipalities had incidence rates below 50 cases per 100,000 inhabitants. On the other hand, 3.2% (*n* = 178) of municipalities had incidence rates above 100 cases per 100,000 inhabitants, accounting for 12.4% (*n* = 28,375) of tuberculosis cases among the elderly during this period (Fig. 3a). A total of 15.1% (*n* = 843) of municipalities were classified in Q1 (high-high), spanning 21 Brazilian states. Together, these municipalities registered 25.4% (*n* = 58,030) of tuberculosis cases in the elderly during the period. Additionally, 78.3% (*n* = 660) of these municipalities were located in the North and Northeast regions (Fig. 3b).

**Fig. 4:**
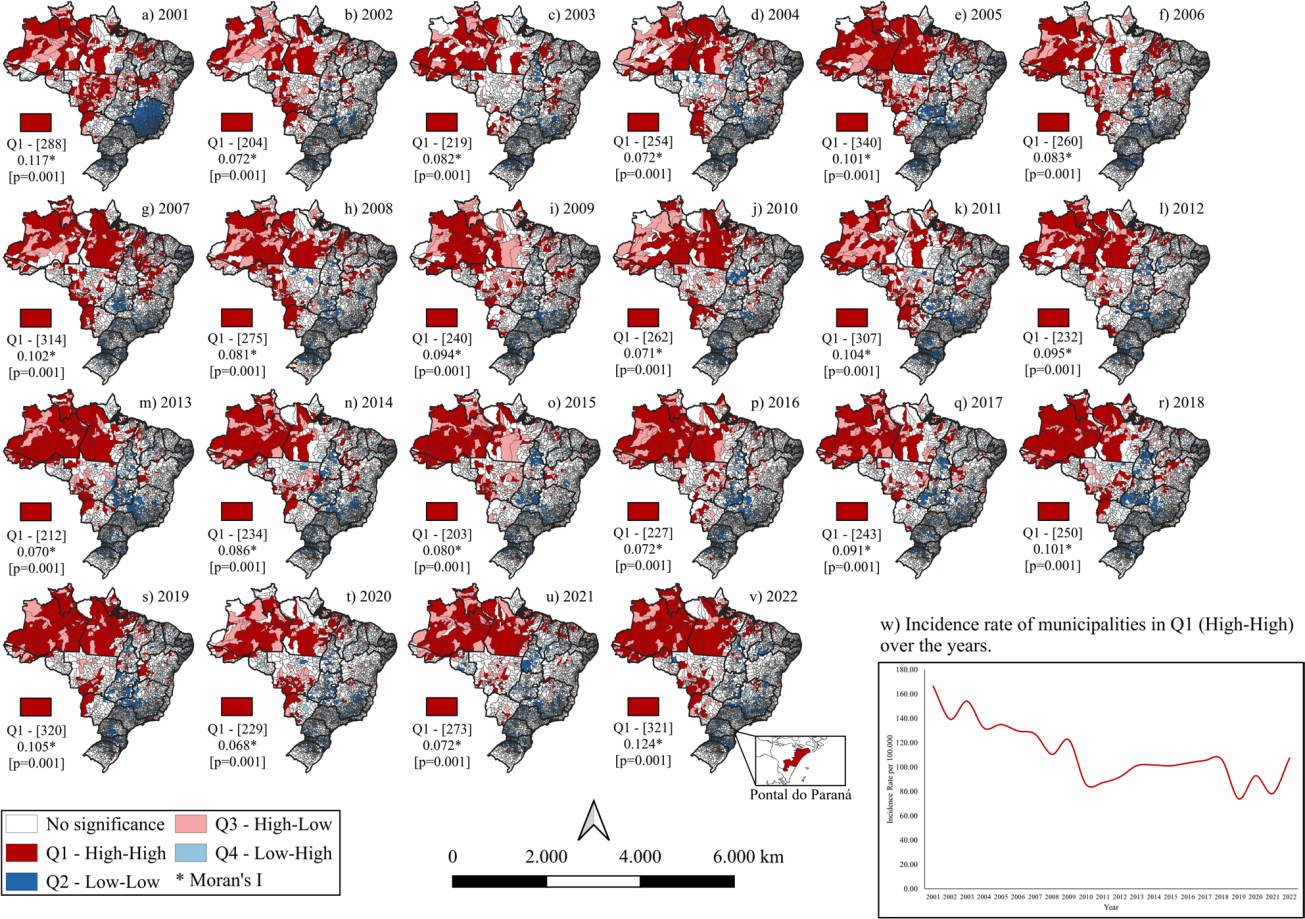
Annual variation of high-high (Q1) clusters of tuberculosis among older adults, Brazil, 2001–2022.The figure demonstrates the evolution of spatial dependence, with a temporary reduction in 2020 coinciding with the COVID-19 pandemic

Between 2001 and 2022, the number of municipalities in Q1 varied from 288 to 321, representing an increase of 11.4%. The predominant regions in 2001 were the North, Northeast, and Central-West, representing 27.1% (*n* = 78), 49.3% (*n* = 142), and 21.2% (*n* = 61) of municipalities in the quadrant, respectively. In 2022, the North and Northeast regions stood out with increases in Q1 municipalities to 48.0% (*n* = 154) and 35.8% (*n* = 115), respectively, while the Central-West region decreased to 10.9% (*n* = 35). The South region, which had no high-high municipalities in 2001, had only the city of Pontal do Paraná classified as such in 2022. It was also observed that in 2020 there was a 28.4% reduction in Q1 municipalities (*n* = 229) compared to the previous year, following a progression from 2015 to 2019 of *n* = 203; 227; 243; 250; and 320 municipalities, respectively (Fig. 4).

When calculating the tuberculosis incidence rate among elderly municipalities in Q1 by year, it was observed that despite the increase in the number of municipalities in this quadrant, the incidence rate decreased from 166.37 per 100,000 inhabitants in 2001 – the highest rate in the analyzed period – to 107.38 per 100,000 inhabitants in 2022, with 2019 standing out as the year with the lowest incidence rate of 73.93 per 100,000 inhabitants (Fig. 4).

## Discussion

This study analyzed selected sociodemographic and clinical indicators, as well as the temporal and spatial trends of tuberculosis cases among the elderly population in Brazil between 2001 and 2022. The findings indicate that most affected individuals were men, aged between 60 and 69 years, Black and Brown people, and those with low or no education. There was a reduction in tuberculosis incidence among the elderly in Brazil and across all regions during the study period, with emphasis on the Central-West region. The North region, despite showing a decreasing trend, stood out for its high incidence rates and the presence of high-risk spatial clusters.

The higher proportion of tuberculosis cases among individuals classified as Black and Brown should be interpreted in light of the official Brazilian race/skin color classification, which is based on self-reported categories and reflects socially constructed identities rather than biological distinctions [15, 16]. Accordingly, these disparities do not indicate biological susceptibility, but instead reflect structural social inequities. National studies have shown that tuberculosis incidence in Brazil is strongly associated with social vulnerability, residential segregation, and unequal access to health services, disproportionately affecting Black and Brown populations [17]. In this context, race/skin color functions as a proxy for structural racism and social vulnerability, influencing exposure, timely diagnosis, and continuity of care [16].

In addition to racial and social inequalities, sex-based differences also shape the epidemiology of tuberculosis among older adults. The predominance of men among the affected may reflect the global male predominance pattern in TB morbidity [18, 19] and is also evident in Brazil, where the male-to-female ratio for pulmonary tuberculosis cases is approximately 2.25:1 [20]. This pattern is influenced not only by biological factors, but also by social and structural determinants, including lower utilization of preventive health services, delayed health-seeking behavior, and greater exposure to occupational risk environments, which together contribute to delayed diagnosis and increased risk among men [21–24]. The predominance of pulmonary forms further amplifies transmission potential, given their high infectivity [25].

Although sex, race/skin color, and education level are recognized as interrelated social determinants of tuberculosis risk, combined analyses of these variables were not performed in this study. This decision was based on the ecological design, the use of aggregated secondary data, and the high proportion of missing or incomplete information when stratifying cases across multiple sociodemographic dimensions, which could compromise statistical stability. Moreover, the study focused on describing population-level spatiotemporal patterns rather than individual-level risk profiles, thereby minimizing the risk of ecological fallacy.

Temporal trend analysis revealed a marked decline in tuberculosis incidence among older adults until approximately 2012–2014, followed by attenuation thereafter. Nationally, incidence decreased significantly between 2001 and 2012, while the subsequent period showed a smaller and non-significant decline, resulting in an overall downward trend. Although this pattern was observed across all macro-regions, there was heterogeneity in the timing and magnitude of inflection points, with the South maintaining a more sustained decline over time.

The initial decline in tuberculosis incidence among older adults likely reflects the consolidation of control strategies in Brazil, including expanded primary health care coverage and improved access to diagnosis and treatment. The subsequent attenuation of this decline may be partly related to changes in diagnostic practices, particularly the introduction and national expansion of the Xpert MTB/RIF assay from 2013 onward. Evidence from a nationwide intervention time-series analysis indicates that Xpert implementation was associated with increased tuberculosis case notification, reflecting improved diagnostic sensitivity rather than a true epidemiological worsening, especially among populations with atypical or paucibacillary presentations, such as older adults [26, 27].

Population aging, the accumulation of comorbidities, persistent social vulnerability, and disruptions associated with the COVID-19 pandemic may also have contributed to sustaining incidence levels in the later period [5, 9, 17, 28]. Older adults, particularly those living with multiple chronic conditions, face increased susceptibility to infection and barriers to timely diagnosis and continuity of care [12]. Together, these factors suggest a multifactorial explanation for the observed change in trend and highlight the need for strategies tailored to older adults and to regions with persistent transmission.

The data also indicate a higher pattern of tuberculosis morbidity among elderly people in the most vulnerable areas of Brazil. A Brazilian study showed that between 1998 and 2019, there were improvements in self-reported health and access to health services among the elderly population in Brazil. However, these advances were unevenly distributed: low-income elderly individuals continued to exhibit poorer health indicators and reduced access to medical consultations, with a greater need for emergency care. These inequalities are exacerbated in the North and Northeast regions, where health service coverage and quality remain more limited [29].

This pattern may be related to socioeconomic conditions and access to healthcare, as social vulnerability is a recognized determinant of tuberculosis in older adults [30, 31]. Evidence from endemic municipalities in the Northeast indicates that lower household income is associated with increased tuberculosis incidence [32]. In contrast, social protection policies implemented in Brazil, particularly income transfer programs such as Bolsa Família and broader initiatives like Fome Zero, have been associated with improvements in living conditions, food security, and access to health services, as well as better tuberculosis-related outcomes, including higher cure rates, lower treatment abandonment, and reduced mortality [33–35]. Although not specifically designed to target tuberculosis or older adults, these policies may have contributed to the decline observed in the earlier period of the series. However, fluctuations in coverage and recent discontinuities appear to have limited their protective effects, particularly in historically vulnerable territories.

Despite these advances, the benefits of social protection policies appear to have been insufficient to sustain a continuous decline in tuberculosis incidence among older adults over time. Until 2012, tuberculosis incidence in this population followed the downward trend observed in the overall Brazilian population, reflecting progress in disease control policies. However, from that point onward, incidence among older adults stabilized, contrasting with the national trend and anticipating the subsequent reversal observed in the general population after 2015 [36].

Socioeconomic and quality-of-life disparities among older adults likely contribute to these temporal and spatial patterns. Global evidence indicates that higher levels of human development are associated with lower tuberculosis incidence, with each one-point increase in the Human Development Index corresponding to an 11.0% reduction in disease incidence [37]. In Brazil, this inverse relationship is particularly evident in the North and Northeast regions, which present the lowest HDI levels, while national studies also demonstrate a strong association between tuberculosis incidence and social vulnerability, especially in relation to Human Capital and Income and Work components of the Social Vulnerability Index [17, 38].

Even within regions with more favorable socioeconomic indicators, important intra-regional heterogeneity was observed. The identification of a high-risk spatial cluster in Pontal do Paraná demonstrates that tuberculosis among older adults may persist in specific municipalities despite the overall declining trend in the South region. This finding underscores that local socioeconomic characteristics and the organization of health services can influence tuberculosis transmission at the municipal level, reinforcing the importance of context-specific analyses when interpreting spatial patterns.

In contrast, the North region has historically been the most affected by tuberculosis in Brazil [39]. The region concentrates the highest levels of multidimensional poverty in the country, affecting 48.4% of its population, with marked deprivation in education, income, and housing conditions [40]. Additionally, weaknesses in Primary Health Care coverage, characterized by lower population coverage and pronounced territorial inequalities in the distribution of PHC teams, compromise surveillance, early diagnosis, active case finding, and continuity of care [41]. The persistence and spatial stability of high-risk clusters in this region likely reflect long-standing social exclusion, poor living conditions, and structural barriers to healthcare access, which sustain continuous chains of transmission, particularly in settings marked by unplanned urbanization and poverty [42].

Moreover, biological factors may also contribute to this epidemiological pattern. A genomic study identified the presence of rare and highly diverse strains of the Central Asian (CAS) genotype of lineage 3 of *Mycobacterium tuberculosis* circulating in the Northern region, particularly in the state of Pará. These strains formed a distinct phylogenetic cluster and were associated with resistance profiles, suggesting local adaptation and a higher potential for dissemination [43]. These factors may contribute to the persistence of areas with a high disease burden and highlight the importance of integrating genomic surveillance with traditional epidemiological monitoring [44].

The Southern region is the second-highest in Brazil in terms of the proportion of people over 60 years of age (17.6%) [9]. Despite this, the region stood out for the absence or low number of municipalities classified as Q1 (high–high) between 2001 and 2022, in contrast to the North and Northeast, which concentrated the highest number of municipalities in this quadrant. This divergence suggests that the burden of tuberculosis among older adults is not solely determined by population aging, but also by contextual and functional factors.

In this regard, the South presents the lowest proportion of older adults dependent on instrumental activities of daily living (IADL) (16.7%), whereas substantially higher proportions are observed in the North and Northeast [45]. IADL limitations have been associated with poorer health outcomes and increased susceptibility to communicable respiratory diseases among older adults (IRR = 1.97) [46–48].

The reduction in the number of municipalities classified as high–high (Q1) clusters in 2020 is likely related to the well-documented underdiagnosis of tuberculosis during the COVID-19 pandemic, a phenomenon observed globally with repercussions in subsequent years [49–51], which influenced observed spatial patterns. In Brazil, tuberculosis diagnoses in the general population decreased by 8.3% in 2020 compared with expected figures, alongside a 4.0% increase in treatment abandonment between 2019 and 2020 [28, 52].

Older adults, particularly those in a state of frailty, were disproportionately affected by pandemic-related disruptions in healthcare services. Reduced access to care, diagnostic delays, and constraints on hospital capacity hindered active case finding and timely tuberculosis treatment, especially in already vulnerable regions [53–55]. These disruptions likely contributed to underreporting and to the temporary changes in tuberculosis incidence and spatial patterns observed during and after the pandemic peak.

It is important to highlight that this study used secondary data from SINAN, which, although essential for national disease surveillance, presents operational limitations, including potential underreporting, incomplete variables, and regional variations in data quality. These factors may affect the accuracy of incidence estimates and the identification of high-risk spatial patterns [56]. Additionally, changes in the number and configuration of municipalities over the study period may have introduced minor inconsistencies in spatial units, potentially influencing the comparability of long-term municipal-level analyses, despite the use of official IBGE territorial boundaries.

In light of the findings, we recommend regionally tailored intervention strategies, with a focus on historically vulnerable areas with a higher disease burden. Municipalities with persistent clusters of high incidence should be prioritized for active surveillance, identification of individuals with respiratory symptoms, and timely diagnosis. Moreover, integration with elderly care networks and intersectoral social protection policies may help address the inequalities that sustain the persistence of tuberculosis among older adults.

## Conclusion

In conclusion, tuberculosis among older adults showed a decreasing trend in Brazil between 2001 and 2022, although with a heterogeneous spatial distribution and persistent concentration in the North and Northeast regions. The predominant profile of affected individuals reinforces the association between social vulnerability and increased risk of illness. These findings underscore the importance of public policies that integrate epidemiological surveillance and equitable access to health services, especially in the context of population aging and regional inequalities in the country. Future studies should further investigate the social and contextual determinants of tuberculosis in the elderly population, incorporating socioeconomic, environmental, and healthcare access variables.

## Data Availability

The datasets analyzed during the current study are publicly available at DATASUS ([http://tabnet.datasus.gov.br/cgi/tabcgi.exe?sinannet/cnv/tubercbr.def] (http://tabnet.datasus.gov.br/cgi/tabcgi.exe?sinannet/cnv/tubercbr.def)).
